# Supplementary material for: Nestin+ Mesenchymal Precursors Generate Distinct Spleen Stromal Cell Subsets and Have Immunomodulatory Function
Source: Int J Mol Sci. 2022 Oct 5;23(19):11819. doi: 10.3390/ijms231911819 (PMC9569994; doi:10.3390/ijms231911819)
Supplement: Supplementary file 1 [file ijms-23-11819-s001.zip › ijms-1872474-supplementary.pdf]

Supplementary Materials:

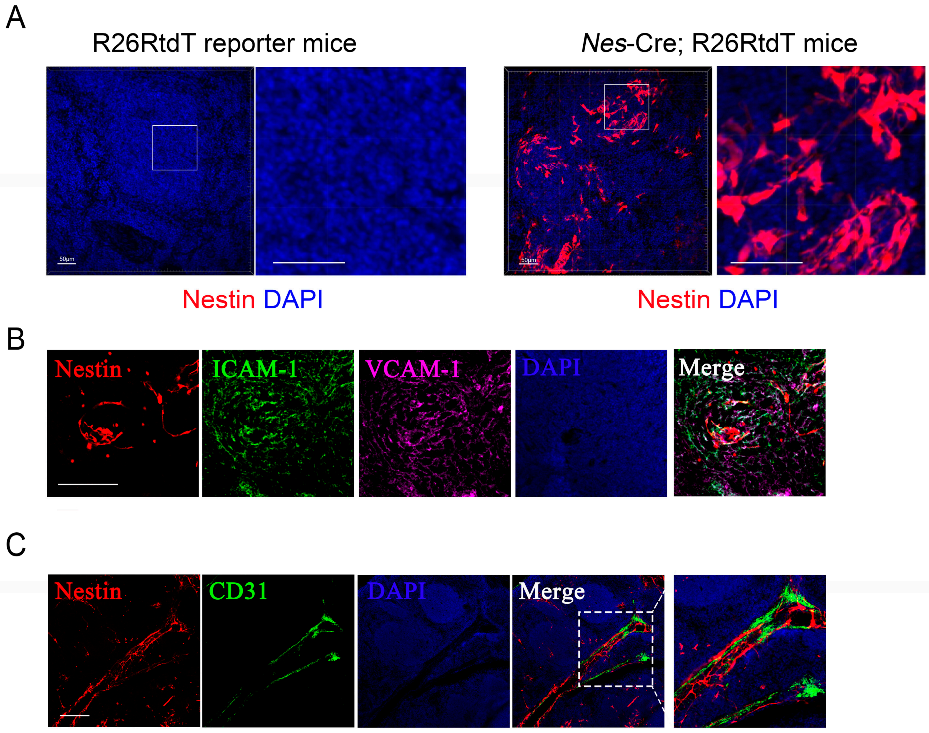

Figure S1. Characterization of Nestin expression in the spleen. (A) Characterization of Nestin expression in the spleen via *R26RtdT* reporter mice and *Nes-Cre; R26RtdT* mice (red). Nuclei were counterstained with DAPI (blue). Scale bar, 50 μm. (B) Immunohistochemical analyses showed the colocalization of Nestin (red) with ICAM-1 (green) and VCAM-1 (purple) in the spleen. Nuclei were counterstained with DAPI (blue). Scale bar, 50 μm. (C) Immunohistochemical analyses of the colocalization between Nestin (red) and CD31 (green) in the spleen. Nuclei were counterstained with DAPI (blue). Scale bar, 50 μm.

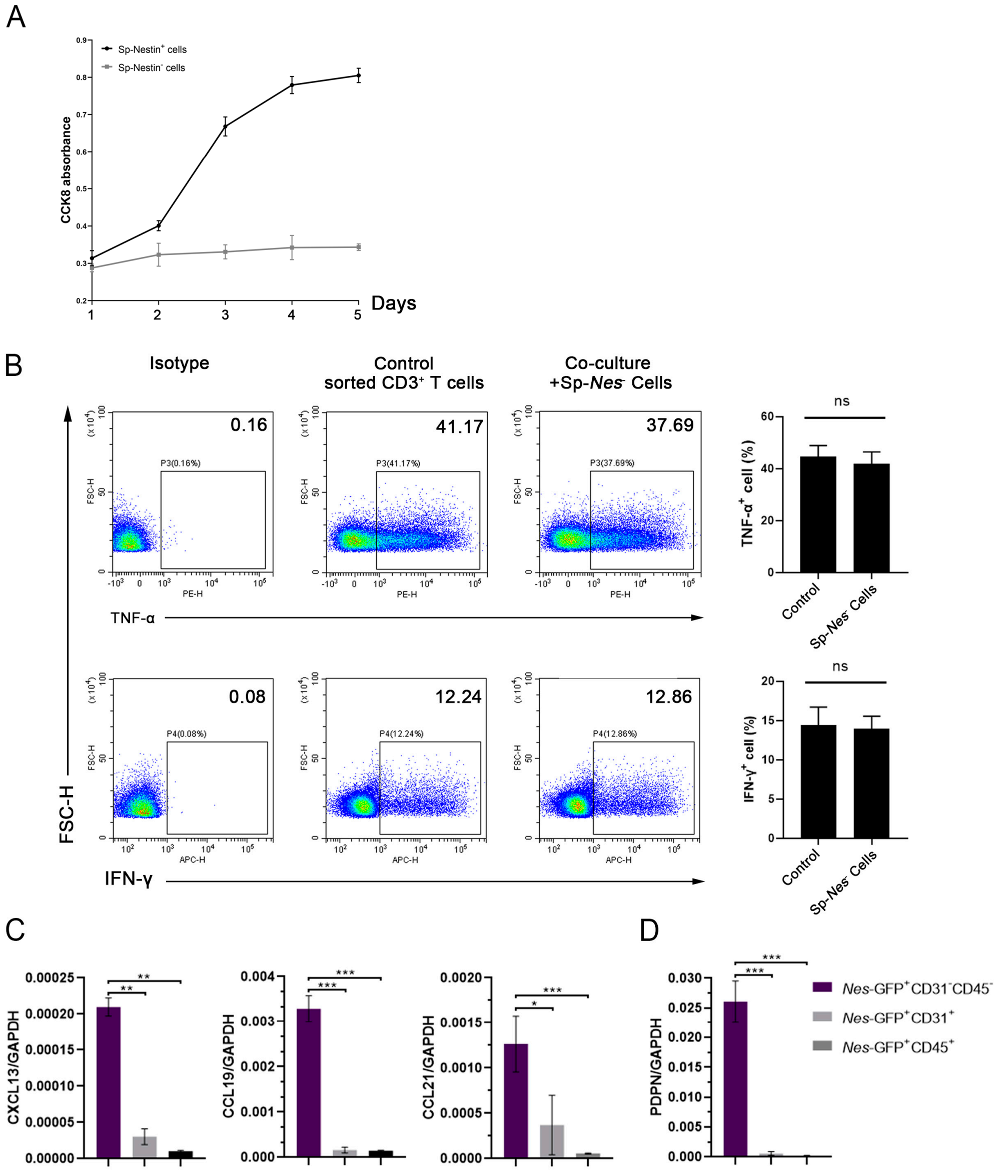

Figure S2. The proliferation ability and immunosuppressive properties of *Nes*-GFP⁻ cells and *Nes*-GFP⁺ cells and characterization of *Nes*-GFP⁺ cells from the spleen. (A) The proliferation rates of the spleen *Nes*-GFP⁻ cells and *Nes*-GFP⁺ cells were assessed using the CCK8 assay and were shown to be different. n=3. Data are expressed as the mean ± SEM. (B) *Nes*-GFP⁻ cells were cocultured with sorted $CD3^+$ T cells, and the pro-inflammatory cytokine TNF-α and IFN-γ secretion of T cells were evaluated by flow cytometry. n=3. Data are expressed as the mean ± SEM. ns, no significant difference. (C, D) qRT-PCR analysis of *Nes*-$GFP^+CD31^-CD45^-$, *Nes*-GFP^+CD31^+, *Nes*-GFP^+ $CD45^+$ cell expressions in the spleen. * $p < 0.05$, ** $p < 0.01$, *** $p < 0.001$.

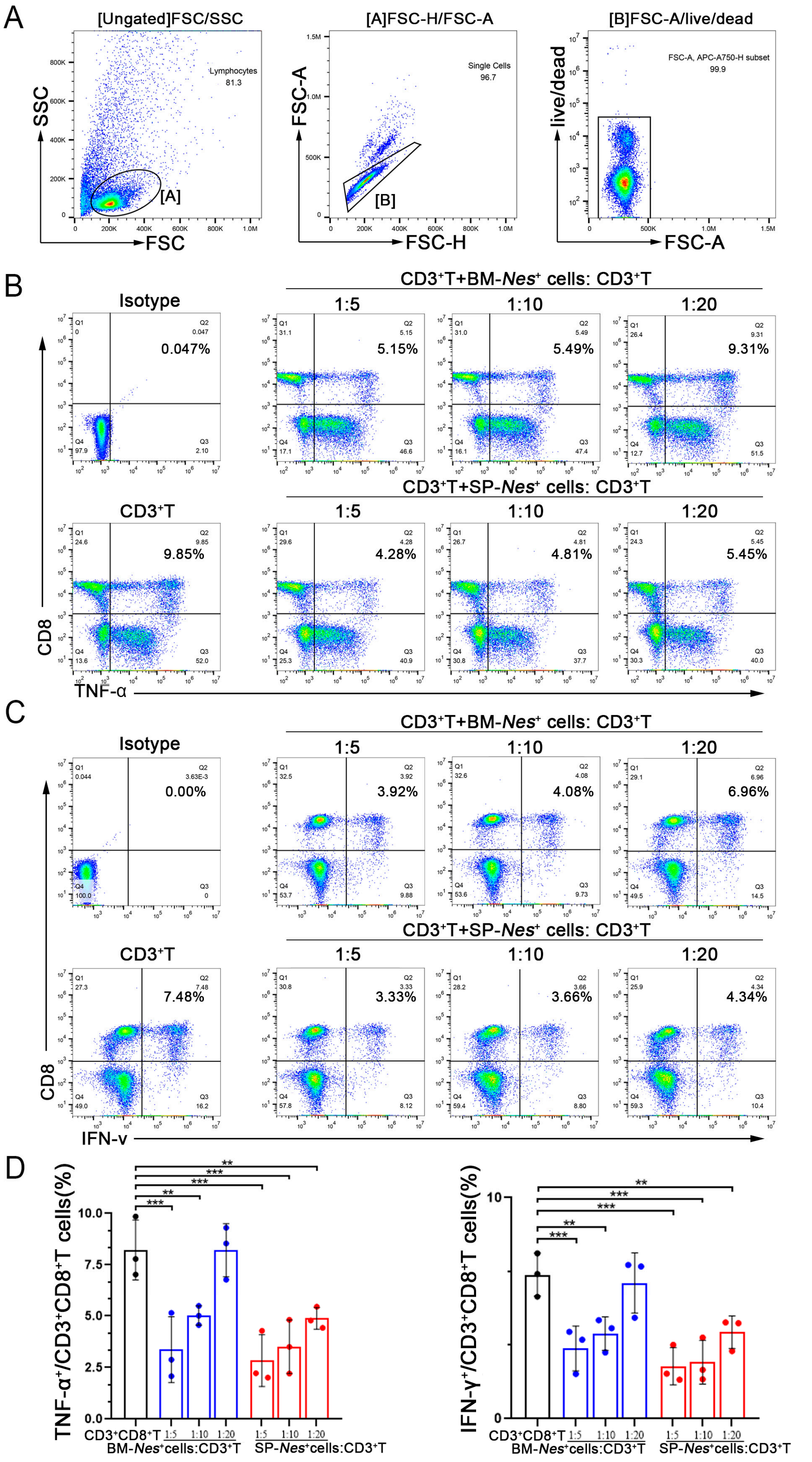

Figure S3. Immunosuppressive properties of *Nes*-GFP⁺ cells (T cells pro-inflammatory cytokine suppression assay). (A) Sorted T cells were collected and stained with Live/Dead before further analysis by flow cytometry. Data show representative results of the staining. (B) *Nes*-GFP⁺ cells were cocultured with sorted CD3⁺ T cells, and the pro-inflammatory cytokine TNF-*α* secretion of CD3⁺CD8⁺T cells were evaluated by flow cytometry. Representative plots of TNF-*α* production by T

cells at different ratio. n=3. (C) *Nes*-GFP$^+$ cells were cocultured with sorted CD3$^+$ T cells, and the pro-inflammatory cytokine IFN-γ secretion of CD3$^+$CD8$^+$T cells were evaluated by flow cytometry. Representative plots of IFN-γ production by T cells at different ratio. n=3. (D) Bar graphs show the inhibition rate of TNF-α and IFN-γ producing CD3$^+$CD8$^+$ T cells after coculture with *Nes*-GFP$^+$ cells. Data represent mean values ± SD of three independent experiments. **P < 0.01, ***P< 0.001.

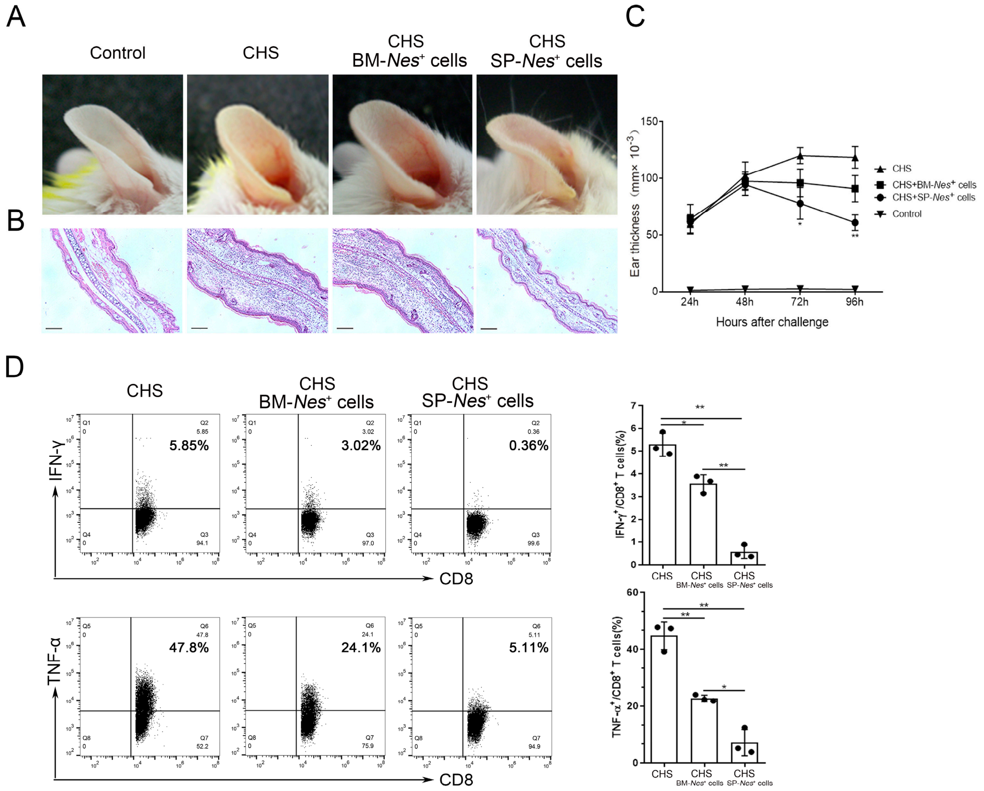

Figure S4. Injection of *Nes*-GFP⁺ cells attenuated CHS. (A-C) Therapeutic efficacy evaluation of CHS model, including (A) Representative photos of ears from mice in different groups, (B) Representative images of H&E-stained ear samples from mice in different groups. Scale bars, 100 μm, (C) Ear thickness was measured in the different experimental groups at the indicated times post challenge. (D) Representative plots of IFN-γ and TNF-α producing $CD8^+$ T cells within cervical LN cells and bar chart showing the quantification. * $p < 0.05$, ** $p < 0.01$.

Table S1. Sequence of Specific Primers Used for qRT-PCR Analysis:

Gene	Forward Sequence (5′-3′)	Reverse Sequence (5′-3′)
GAPDH	ACCACAGTCCATGCCATCAC	TCCACCACCCTGTTGCTGTA
Nestin	AGGAGAAGCAGGGTC-TACAGAG	AGTTCTCAGCCTCCAG-CAGAGT

Table S2. Antibodies for Flow Cytometry.

Antigen	Source	Dilution	Identifier
Anti-mouse CD106 (AF647)	Biolegend	1:100	105712
Anti-mouse CD29 (PE)	BD Biosciences	1:100	562801
Anti-mouse PDPN (ef660)	eBioscience	1:100	50-5381-80
Anti-mouse CD90 (PE)	eBioscience	1:100	12-0902-81
Anti-mouse CD31 (PE)	BD Biosciences	1:100	553373
Anti-mouse CD45 (APC)	Invitrogen	1:100	MCD4505
Anti-mouse CD11c (PE)	BD Biosciences	1:100	561044
Anti- mouse CD3 (V450)	BD Biosciences	1:100	560804
Anti- mouse CD8 (APC)	BD Biosciences	1:100	553035
Anti-mouse IFN-γ (PE-Cy7)	BD Biosciences	1:100	557649
Anti-mouse TNF-α (PE)	BD Biosciences	1:100	554419
Anti-mouse CD25 (APC)	eBioscience	1:100	17-0251-81
Anti-mouse CD69 (PE)	Biolegend	1:100	104507
Near-IR fluorescent reactive dye	Invitrogen	1:1000	L34967
CellTrace™ Yellow	Invitrogen	1:1000	C34573
AF647 IgG2a, κ Isotype	Biolegend	1:100	RTK2758
PE IgG2, λ1 Isotype	BD Biosciences	1:100	553965
APC IgG Isotype	Invitrogen	1:100	17-4914-81
PE IgG2a, κ Isotype	Invitrogen	1:100	12-4321-80
APC IgG2b, κ Isotype	Invitrogen	1:100	PA5-33192
PE IgG Isotype	Invitrogen	1:100	12-4888-83
V450 IgG Isotype	Invitrogen	1:100	48-4914-80
APC IgG2a, κ Isotype	BD Biosciences	1:100	551139
PE-Cy7 IgG1, κ Isotype	Biolegend	1:100	RTK2071
PE IgG1 Isotype	Biolegend	1:100	MRG1-58
APC IgG1 κ Isotype	Invitrogen	1:100	17-4301-82
PE IgG Isotype	Biolegend	1:100	HTK888

Table S3. Antibodies for Immunofluorescence Staining.

Antigen	Source	Dilution	Identifier
Rat anti-mouse ER-TR7	AbD Serotec	1:100	MCA2402
Rat anti-mouse CD35	BD Biosciences	1:100	553816
Rat anti-mouse MadCAM-1	Abcam	1:100	Ab254539
Anti-mouse ICAM-1 (FITC)	Invitrogen	1:100	11-0541-82
Anti-mouse VCAM-1 (AF647)	Biolegend	1:100	105712
Anti-mouse CD31 (FITC)	Invitrogen	1:100	11-0311-82
Anti-mouse CD3 (AF647)	Biolegend	1:100	152320
Anti-mouse B220 (AF594)	Biolegend	1:100	103254
Goat Anti-rat IgG Alexa 594	Invitrogen	1:500	A11007
Goat Anti-rat IgG Alexa 647	Invitrogen	1:500	A21247
